# Continuous Electrical Monitoring in Patients with Arrhythmic Myocarditis: Insights from a Referral Center

**DOI:** 10.3390/jcm10215142

**Published:** 2021-11-01

**Authors:** Giovanni Peretto, Patrizio Mazzone, Gabriele Paglino, Alessandra Marzi, Georgios Tsitsinakis, Stefania Rizzo, Cristina Basso, Paolo Della Bella, Simone Sala

**Affiliations:** 1 Department of Cardiac Electrophysiology and Arrhythmology, IRCCS San Raffaele Scientific Institute, 20132 Milan, Italy; mazzone.patrizio@hsr.it (P.M.); paglino.grabriele@hsr.it (G.P.); marzi.alessandra@hsr.it (A.M.); gio.tsitsi@yahoo.gr (G.T.); dellabella.paolo@hsr.it (P.D.B.); sala.simone@hsr.it (S.S.); 2 Myocarditis Disease Unit, IRCCS San Raffaele Scientific Institute, 20132 Milan, Italy; 3 School of Medicine, San Raffaele Vita-Salute University, 20132 Milan, Italy; 4 Department of Cardiac Thoracic Vascular Sciences and Public Health, Cardiovascular Pathology, Padua University, 35128 Padua, Italy; s.rizzo@unipd.it (S.R.); cristina.basso@unipd.it (C.B.)

**Keywords:** myocarditis, arrhythmias, telemonitoring, implantable cardioverter defibrillator, implantable loop recorder, Holter ECG

## Abstract

Background. The incidence and burden of arrhythmias in myocarditis are under-reported. Objective. We aimed to assess the diagnostic yield and clinical impact of continuous arrhythmia monitoring (CAM) in patients with arrhythmic myocarditis. Methods. We enrolled consecutive adult patients (*n* = 104; 71% males, age 47 ± 11 year, mean LVEF 50 ± 13%) with biopsy-proven active myocarditis and de novo ventricular arrhythmias (VAs). All patients underwent prospective monitoring by both sequential 24-h Holter ECGs and CAM, including either ICD (*n* = 62; 60%) or loop recorder (*n* = 42; 40%). Results. By 3.7 ± 1.6 year follow up, 45 patients (43%) had VT, 67 (64%) NSVT and 102 (98%) premature ventricular complexes (PVC). As compared to the Holter ECG (average 9.5 exams per patient), CAM identified more patients with VA (VT: 45 vs. 4; NSVT: 64 vs. 45; both *p* < 0.001), more VA episodes (VT: 100 vs. 4%; NSVT: 91 vs. 12%) and earlier NSVT timing (median 6 vs. 24 months, *p* < 0.001). The extensive ICD implantation strategy was proven beneficial in 80% of the population. Histological signs of chronically active myocarditis (*n* = 73, 70%) and anteroseptal late gadolinium enhancement (*n* = 26, 25%) were significantly associated with the occurrence of VTs during follow up, even in the primary prevention subgroup. Conclusion. In patients with arrhythmic myocarditis, CAM allowed accurate arrhythmia detection and showed a considerable clinical impact.

## 1. Introduction

Continuous arrhythmia monitoring (CAM) via implantable devices represents the gold standard for the detection of arrhythmias under many medical conditions [1,2]. In fact, in contrast to non-continuous monitoring by either Holter ECGs or short-term external devices [3], CAM allows the continuous and potentially life-long evaluation of cardiac electrical activity. In myocarditis, CAM may be useful to fill in relevant knowledge gaps on the incidence, type and burden of arrhythmias [4,5]. This is clinically important since ventricular arrhythmias (VAs) and bradyarrhythmias (BAs) constitute life-threatening complications of myocarditis [6,7]. Furthermore, the incidence of atrial fibrillation (AF) and other supraventricular arrhythmias (SVAs) is unknown in this setting. To date, no studies have investigated the benefits of CAM application in patients with myocarditis. In fact, indications for implantable cardioverter defibrillators (ICDs) are restricted in this population [5,6] and there is currently no experience about the use of implantable loop recorders (ILRs) as long-term monitoring devices. Because of the episodic nature of arrhythmias, we hypothesized that, even in the myocarditis population, CAM had a superior diagnostic yield compared to even regularly repeated Holter ECGs. In addition, we aimed to assess the appropriateness of the ICD implantation strategy in patients presenting with clinically defined acute myocarditis but heterogeneous histopathological findings.

## 2. Methods

### 2.1. Study Design

**Figure 1:**
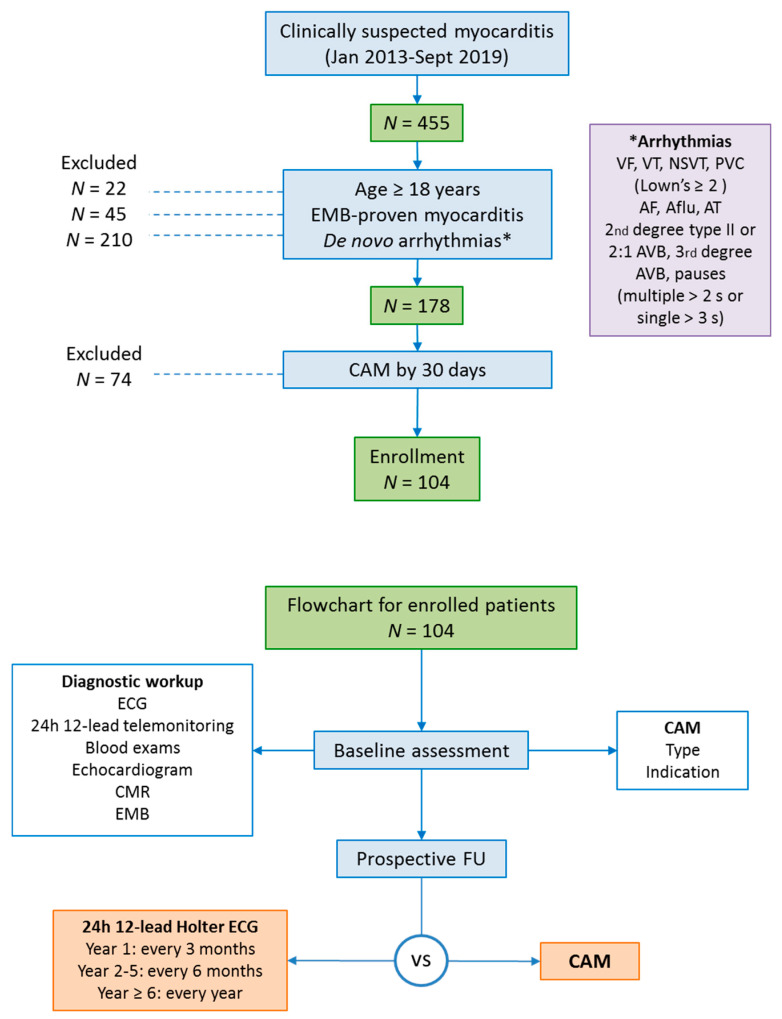
Study flowchart: study design with inclusion criteria is shown. AF = atrial fibrillation; AFlu = atrial flutter; AT = atrial tachycardia; AVB = atrioventricular blocks; CAM = continuous arrhythmia monitoring; CMR = cardiac magnetic resonance; EMB = endomyocardial biopsy; FU = follow up; NSVT = nonsustained ventricular tachycardia; PVC = premature ventricular complexes; VA = ventricular arrhythmia; VF = ventricular fibrillation; VT = ventricular tachycardia.

This was a single-center observational study with a prospective follow up reflecting the experience of a referral center. This study was in compliance with the Declaration of Helsinki and underwent Institutional Review Board approval. The study flowchart is presented in Figure 1. Between January 2013 and January 2019, consecutive patients with arrhythmic myocarditis were enrolled. The following inclusion criteria were applied: (1) age ≥ 18 year; (2) EMB-proven diagnosis of active myocarditis [5]; (3) evidence of previously unknown (or de novo) arrhythmias at index hospitalization; and (4) a CAM strategy started within 30 days from myocarditis diagnosis.

As part of the baseline diagnostic work-up, all patients underwent complete blood exams, continuous 12-lead ECG telemonitoring, transthoracic echocardiogram and cardiac magnetic resonance (CMR).

### 2.2. Definitions

Arrhythmias were defined based on updated standards [8,9,10] and classified into VA, SVA and BA. In detail, VA included ventricular fibrillation (VF), tachycardia (VT), nonsustained VT (NSVT) and grade ≥2 premature ventricular complexes (PVCs) according to Lown’s classification (i.e., >1 PVC/min or >30 PVC/h) [11]; SVA included AF, atrial flutter and atrial tachycardia; BA included 2nd degree type II, 2:1, or 3rd degree atrioventricular blocks (AVBs) and pauses >3 s. Further definitions, including details concerning VA characterization, are reported in the Supplementary Materials.

Histological signs of fibrosis, cardiac myocyte hypertrophy and nuclear atypia were used to define “chronically active” rather than true “acute” myocarditis [12,13].

### 2.3. CAM Selection

In the absence of clear guideline recommendations for patients with chronically active myocarditis [5,6,7], the choice between ICD and ILR was patient-tailored and guided the experience of a referral center for arrhythmia management [14]. In detail, the following putative risk factors were identified a priori as markers of arrhythmic risk: (1) left ventricular ejection fraction (LVEF) < 35% at baseline echocardiogram; (2) non-lymphocytic histotypes, namely cardiac sarcoidosis and giant cell myocarditis; (3) 2nd or 3rd degree AVB; (4) fast (>180 bpm for at least 10 beats) or recurrent (>3 episodes at telemonitoring) NSVT despite antiarrhythmic therapy; (5) induction of VT or VF at baseline programmed ventricular stimulation (PVS) when applicable; (6) extensive areas of either late gadolinium enhancement (LGE) at CMR (>1 LV wall, or >5 of 17 LV segments) or replacement fibrosis at histology (>50% of tissue samples).

For secondary prevention, the ICD implant was indicated following either VT or VF onset. Otherwise, CAM was proposed to all patients: the decision between the primary prevention ICD and ILR implant was personalized, and guided by the above defined risk factors. Details about CAM programming are reported in the Supplementary Materials.

### 2.4. Follow-Up

All patients underwent prospective follow-up (FU) reassessment [15] through both CAM and 12-lead 24 h Holter ECGs, according to a defined schedule (4/year in the first year; 2/year in years 2–5; and then 1/year). Both in-person and remote monitoring were allowed for CAM, and the arrhythmia timeline was defined by the real event date. The association with symptoms was assessed both by the analysis of manually activated device alerts, and by direct patient interrogation.

### 2.5. Endpoints

VA occurrence, burden and timing—as detected by CAM vs. Holter ECG monitoring—were analyzed as the primary study endpoint. During FU, appropriate ICD interventions (anti-tachycardia pacing or shock) also constituted VT events. The occurrence of other arrhythmias (SVA, BA) constituted the secondary endpoints. In addition, the appropriateness of the ICD implantation strategy was retrospectively evaluated.

### 2.6. Statistical Analysis

SPSS Version 20 (IBM Corp., Armonk, NY, USA) was used for the analysis, and Prism Version 6 (GraphPad Software Inc., La Jolla, CA, USA) was used for graphic presentations. Continuous variables were expressed as the mean and standard deviation, or as median and IQR of 25th to 75th percentiles, depending on the distribution of data. Accordingly, continuous variables were compared by Student’s *t*-test or by Mann–Whitney U-test. Categorical variables, reported as counts and percentages, were compared by the Fisher exact test. Cox regression and Kaplan–Meier curves were used for event rate analyses. Where relevant, 2-sided *p*-values < 0.05 were set as statistically significant. Confidence intervals were set at 95%.

## 3. Results

### 3.1. Baseline Characteristics of the Population

Overall, 104 patients (71% males, mean age 47 ± 11 year) were enrolled, including those with arrhythmic presentation (*n* = 70) and those with arrhythmias detected during in-hospital telemonitoring (*n* = 34). Patients’ complete characteristics are shown in Table 1. Arrhythmias included VAs, SVAs and BAs in 104 (100%), 11 (11%), and 9 patients (9%), respectively. Overall, 19 patients (18%) had LVEF < 35% at presentation. EMB identified 73 cases of chronically active myocarditis (70%) and CMR showed anteroseptal LGE in 26 cases (25%).

### 3.2. CAM Types, Indications and Complications

ICDs were implanted in 62 patients (60%; *n* = 47 for secondary prevention), including dual-chamber (*n* = 48), single-chamber (*n* = 5) and subcutaneous devices (S-ICD, *n* = 9). The remaining 42 patients (40%) underwent ILR implant. The mean number of risk factors was two in ICD carriers and <1 in ILR cases (Table S1). Among the 61 patients undergoing PVS, 25 had VT or VF inducibility and underwent ICD implant (Table S2). Complications were documented in 9/62 ICD carriers (15%) including infection (*n* = 3), catheter dislocation or malfunctioning (*n* = 3), hematoma (*n* = 2) and pneumothorax (*n* = 1). No complications occurred following ILR implants.

### 3.3. Treatment and Follow Up

All patients were discharged on medical treatment, including RAAS-inhibitors (*n* = 87), betablockers (*n* = 96), and either single (*n* = 47) or combined (*n* = 23) antiarrhythmic drug (AAD) therapy (Table S3).

The study FU was 3.7 ± 1.6 year. There were no patients lost to FU. The average number of Holter ECGs per patient was 9.5, and the proportion of missed exams was 3.6% (maximum one exam per patient). Three patients died (end-stage heart failure, *n* = 1; infectious complications of cardiac transplantation, *n* = 1; malignancy, *n* = 1), all of which were ICD carriers (guideline-driven implant in two of them). No patients experienced systemic embolism or hemorrhagic complications.

During FU, CMR was repeated in 40 cases (38%), and its interpretation was limited by susceptibility artifacts in all ICD (*n* = 5) and no ILR carriers (*n* = 35, 28 of whom were proven healed from myocarditis). All devices were replaced following the end-of-life status. No quality-of-life issues were reported by 91% of the device carriers (Table S4).

### 3.4. VA Detection, Burden and Timing

**Figure 2:**
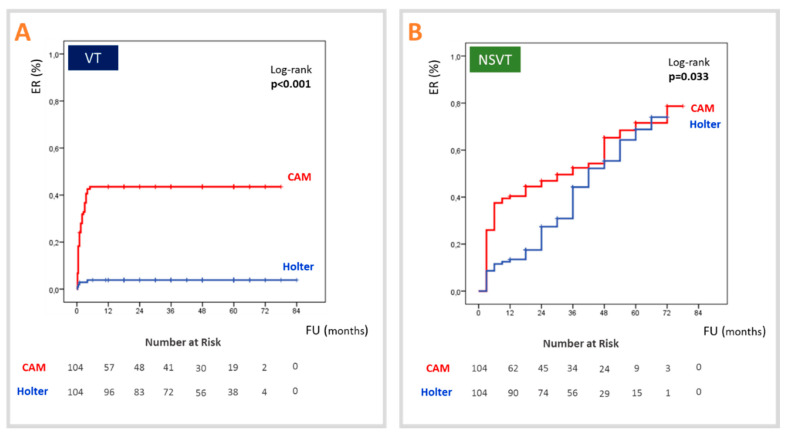
Detection of ventricular arrhythmias by CAM vs. sequential 24 h Holter ECGs. Kaplan–Meier curves are shown for the endpoint of VT (panel **A**) and NSVT (panel **B**). CAM = continuous arrhythmia monitoring (red); ER = event rate; FU = follow up; Holter = 24 h Holter ECG (blue); NSVT = nonsustained ventricular tachycardia; VT = ventricular tachycardia.

During FU, 45 patients (43%) underwent VT episodes including *n* = 3 incessant VTs, *n* = 10 electrical storms (≥3 shocks/24 h) and *n* = 32 paroxysmal VTs only. In 10/45 cases (22%), there was no prior history of VT. In addition, 67 patients (64%) had NSVT and 102 (98%) PVC. Complete data are reported in Table 2. As compared to Holter ECG, CAM identified more patients either with VT (45 vs. 4, *p* < 0.001) or NSVT (64 vs. 45, *p* < 0.001). Kaplan–Meier curves are shown in Figure 2. All VT episodes and most of the NSVT ones were only detected by CAM (Table 2); in addition, CAM allowed earlier NSVT detection (median 6, IQR 3–24 vs. median 24, IQR 9–36 months, respectively, *p* < 0.001). Event rates are shown in Figure S1.

Although an alert for clustered PVC was reported by CAM in 21 cases (21%), PVCs were documented by Holter ECG in 102/102 patients (*p* < 0.001). During FU, CAM showed a significant reduction in VT/NSVT cycle length variability, whereas the Holter ECG documented a progressive prevalence of monomorphic PVC (Figure S2).

### 3.5. Other Arrhythmias

During FU, SVA episodes were documented in 27 patients (26%) including AF in 19 cases (18%). In addition, six patients had BA, mainly second- and third-degree AVB. Complete data are shown in Table 2. Overall, CAM identified more patients either with SVA lasting > 24 h (9 vs. 1, *p* < 0.001), or BA (6 vs. 1, *p* = 0.015) and only missed pauses in the range of 2–3 s. SVA detection by CAM was earlier than that by Holter ECG (22 ± 8 months in 27 patients vs. 36 ± 12 months in 7 patients, respectively, *p* = 0.001).

### 3.6. CAM Type and Indication

Arrhythmia recordings in different CAM subgroups are shown in Table S5. Although most VA occurred in patients following secondary prevention ICD implant, VTs were also documented within primary prevention ICD (10 episodes in *n* = 8 patients) and ILR subgroups (two episodes in two patients).

A FU VT was found in 40/80 patients with putative risk factors vs. 5/24 without putative risk factors (HR 3.8, 95% CI 1.3–11.2, *p* = 0.015). However, there was no a single risk factor capable of predicting the occurrence of a de novo VT (Table 3). Instead, our post hoc analysis identified both anteroseptal LGE distribution pattern at CMR, and signs of chronically active myocarditis at EMB, as significantly associated with the first episode of VT during FU (respectively: 50 vs. 13% and 90 vs. 49%, both *p* < 0.05). Results were confirmed for the whole study cohort, where VT episodes were more common in the chronically active myocarditis and anteroseptal LGE subgroups (respectively: 40/73 vs. 5/31 acute cases, *p* < 0.001; and 16/26 vs. 29/78 inferolateral cases, *p* = 0.04).

### 3.7. Clinical Impact

Guided by CAM for VT episodes and by Holter ECG for high-burden PVCs, 41 patients (39%) underwent transcatheter ablation during FU. Apart from the VT episodes, most FU arrhythmias were asymptomatic (Table S6). Significantly, de novo oral anticoagulants were started in eight SVA patients (8%) including six asymptomatic ILR carriers. An upgrade to dual-chamber ICD was performed in eight cases (8%) including ILR patients (*n* = 5; two for VT and three for NSVT associated with BA), and ICD cases experiencing inappropriate shocks for AF (*n* = 3; two single-chamber ICDs and one S-ICD).

**Figure 3:**
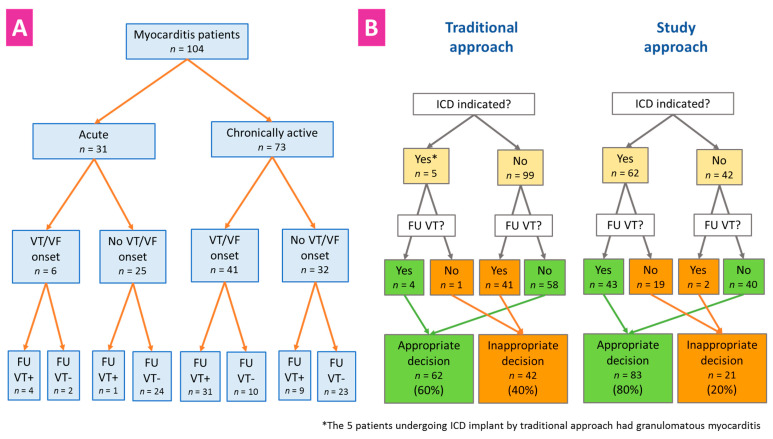
Events by myocarditis stage and implantation strategy. Panel **A**: VT events (VT+) in patients with true acute vs. chronically active myocarditis according to endomyocardial biopsy findings; Panel **B**: Appropriateness of the ICD implantation strategy by application of current guidelines for acute myocarditis (left panel) vs. by multiparametric approach as described in this study (right panel). FU = follow up; ICD = implantable cardioverter defibrillator; VF = ventricular fibrillation; VT = ventricular tachycardia.

Based on the current guideline recommendations [5,6], only the five patients with granulomatous myocarditis (5%) and VT/VF onset would have met the criteria for an early ICD implant. However, among the 99 candidates for an ICD-sparing strategy, 41 (41%) experienced at least one VT episode during FU. By the end of the study, the ICD implantation strategy was appropriate in 80% of the population instead of 60%, resulting from the strict application of the guidelines (Figure 3).

## 4. Discussion

### 4.1. Major Findings

We described the first study aimed at exploring the advantages of CAM as compared to standard Holter ECG monitoring in patients with EMB-proven active myocarditis [5,13] and evidence of arrhythmias at index hospitalization. Remarkably, the comparison between techniques was unbiased since all patients underwent both CAM and Holter monitoring strategies. Despite the considerable number of Holter ECG exams per patient, we showed that CAM was more accurate in both detecting and quantifying most of the clinically impactful arrhythmias. In addition, we showed that despite a uniform clinical presentation with acute myocarditis [5,6], many patients had histopathological signs suggesting chronically active disease [4,14]: in light of the significant association with follow-up VT episodes, an earlier indication of the ICD implant could be considered for the latter ones.

### 4.2. Diagnostic Accuracy for VA

As shown in Table 2, all FU VT episodes were detected by CAM. Compared to Holter ECG, CAM was superior in both identifying patients with VA and detecting total VA episodes. Although more frequently detected by ICDs, VA episodes were also found in a relevant proportion of ILR carriers (Table S5). Conversely, the CAM accuracy in detecting PVCs was remarkably lower compared to Holter ECG, which allowed precise PVC quantification over time [10]. As a relevant guidance for the planning of catheter ablation strategies, the clinical VA morphology requires documentation by 12-lead ECG recording [10,16]. Recently, VA characterization by ECG has also been proposed as a tool to assess the myocardial inflammatory stage [17,18] and identify the suitable candidates for VT ablation [16]. In keeping with myocarditis healing, CAM recordings documented a progressive reduction in VA cycle length variability during follow-up, in parallel with a prevalence of monomorphic PVC by Holter ECG (Figure S2).

### 4.3. Other Arrhythmias

Table 2 shows that CAM was an accurate tool also for diagnosing SVA and BA. Remarkably, most of the long-lasting SVAs were those which were late onset (Figure S1) and asymptomatic (Table S6). In this setting, the CAM-guided anticoagulation strategy [19] was safe since no ischemic or hemorrhagic complications occurred. In turn, advanced AVBs, commonly reported in acute-phase cardiac sarcoidosis [4], were documented even later during FU. Although iatrogenic effects from betablockers and AADs were likely (Table S3), the documentation of both BA and NSVT constituted an indication to ICD upgrading in three ILR carriers (Table S6). Instead, the possible underdiagnosis of BA in transvenous ICD carriers constituted a clinically neglectable issue.

### 4.4. Arrhythmic Risk Estimation

In our study, the indication of ICD was supported by a number of pre-selected risk factors, namely: LVEF < 35% [6,7]; malignant histotypes [4]; major BA [9]; fast/recurrent NSVT [10]; positive PVS [20]; and extensive LGE or myocardial fibrosis [21,22]. Although VT events more commonly occurred in patients with at least one of the above risk factors, none of the candidates were able to predict an adverse outcome in primary prevention. In keeping with prior studies, we identified anteroseptal LGE [23,24,25,26] and histological signs suggesting chronic myocarditis [12,13] as factors associated with adverse arrhythmic outcomes, both in the whole cohort and in patients without malignant VA onset. Results are consistent with recently published data [27]. As suggested by Table 3, mild systolic dysfunction (i.e., LVEF < 50%) may play an additional role for primary prevention risk stratification, as already suggested both in myocarditis and other cardiomyopathies [28,29].

### 4.5. Device Indication and Choice

Overall, our data challenge the uniform application of an ICD-sparing strategy in patients with VA onset and newly diagnosed active myocarditis [5,6]. Actually, our analysis revealed that, despite the clinically acute myocarditis onset, the majority of patients in our cohort had histological signs of chronic myocarditis, as supported by myocardial fibrosis and additional cellular abnormalities [12,13]. In contrast to the truly “acute” myocarditis cases, those with “chronically active” inflammation showed a significantly higher occurrence of VT during FU—even in the absence of granulomatous myocarditis (Figure 3). Our findings indicate that clinical guidelines may benefit from a clear distinction between the scenarios, and we suggest that a multiparametric assessment could be implemented in chronic setting to identify the most suitable candidates for an early ICD implant [14].

As for the device choice, in our experience, dual-chamber ICDs are advisable to minimize the risk of inappropriate shocks by single-lead devices. In turn, since scar-related VA may even occur during the post-inflammatory stage of myocarditis [16,17], the use of wearable cardioverter defibrillators could be undermined by the unpredictable optimal timing for device withdrawal: while life-vests are currently recommended as a bridge for decision making in acute myocarditis [5,30], S-ICDs may constitute a valuable alternative in the chronic setting. Finally, because of a combination of high diagnostic accuracy, general acceptance among patients (Table S4) and CMR feasibility [31,32], we suggest the widespread use of ILRs as optimal diagnostic tools for the remaining low-risk patients with arrhythmic myocarditis [33,34].

### 4.6. Study Limitations

Our study specifically focused on patients with myocarditis and the evidence of VA at the index of hospitalization. Although the arrhythmic population is underinvestigated and clinically demanding [4,5,6,7], results should not be inappropriately generalized to different clinical scenarios. Selection bias related to the center experience [14,33] as well as baseline arrhythmia overdetection due the use of continuous in-hospital telemonitoring should be taken into account. Importantly, CAM choice was conditioned by a number of risk factors that, although reasonable, were not supported by robust evidence—this introduces a bias by indication. Baseline PVS was not performed in all patients, and wearable devices were not hereby investigated. Finally, some differences in arrhythmia detection capability should be considered for ICDs (unable to detect BA unless permanent pacing is needed) and for single-chamber and subcutaneous devices (which may be less reliable in differentiating SVA and VA subtypes). Larger prospective multicenter studies are needed to validate our findings and improve patient selection for each device type at different inflammatory stages [16,17,18].

## 5. Conclusions

In patients with arrhythmic myocarditis, CAM was a clinically useful tool to detect arrhythmias and guide relevant therapeutical decisions. As compared to sequential Holter ECGs, CAM allowed an earlier detection and greater diagnostic yield for most arrhythmias. As a complementary tool, Holter ECG allowed PVC quantification and morphology characterization. Based on our findings, efforts are needed to identify patients with chronically active myocarditis, as well as those with anteroseptal LGE at CMR, who may benefit from an earlier ICD implant. In low-risk patients, ILR was a feasible and sensitive diagnostic tool, allowing also to monitor myocarditis evolution by informative CMR. Prospective controlled trials including appropriate myocarditis staging and a uniform implantation strategy are needed, to improve the arrhythmic risk stratification and patient selection for different device types.

## Figures and Tables

**Table 1:** Baseline characteristics of the population.

Parameter	Units	Total *N* = 104
Clinical data		
Age (year)	Mean ± SD	47 ± 11
Sex (male)	N (%)	74 (71)
Caucasian	N (%)	98 (94)
Presentation		
ACS-like	N (%)	14 (13)
HF	N (%)	20 (19)
Arrhythmias	N (%)	70 (67)
Family history of SCD/CMP	N (%)	6 (6)
Fever in last 30 days	N (%)	35 (34)
Syncope	N (%)	37 (36)
Palpitation	N (%)	72 (69)
Chest pain	N (%)	25 (24)
Dyspnea	N (%)	40 (38)
NYHA class	Median (IQR)	1 (1–2)
Blood exams		
WBC (103/mm^3^)	Mean ± SD	8.5 ± 3.5
Neutrophils (%)	Mean ± SD	63 ± 12
CRP (mg/L; n.v. < 6)	Median (IQR)	3.2 (1.5–8.8)
T-Tn (ng/L; n.v. < 14)	Median (IQR)	40 (9–456)
NTproBNP (pg/mL; n.v. < 125)	Median (IQR)	198 (82–843)
ECG		
HR (min^−1^)	Mean ± SD	76 ± 22
PQ (ms)	Mean ± SD	173 ± 28
QRS (ms)	Mean ± SD	103 ± 21
QTc (ms)	Mean ± SD	423 ± 34
Abnormal T waves	N (%)	59 (57)
Abnormal ST	N (%)	30 (29)
Telemonitoring		
Total VA	N (%)	104 (100)
PVC	N (%)	102 (98)
PVC daily number	Median (IQR)	1201 (209–3390)
NSVT	N (%)	43 (41)
VT	N (%)	39 (38)
VF	N (%)	8 (8)
Total SVA	N (%)	11 (11)
AF	N (%)	9 (9)
Atrial flutter	N (%)	1 (1)
Atrial tachycardia	N (%)	4 (4)
NSAT	N (%)	5 (5)
Total BA	N (%)	9 (9)
Pauses > 3 s	N (%)	3 (3)
1st degree AVB	N (%)	15 (14)
2nd degree AVB Mobitz 1	N (%)	1 (1)
2nd degree AVB Mobitz 2	N (%)	2 (2)
2nd degree AVB 2:1	N (%)	1 (1)
3rd degree AVB	N (%)	6 (6)
Echocardiogram		
LV EDVi (mL/m^2^)	Mean ± SD	68 ± 20
LV EF (%)	Mean ± SD	50 ± 13
Regional WMA	N (%)	59 (57)
E/E’	Mean ± SD	8 ± 3
RV EDD (mm)	Mean ± SD	32 ± 4
TAPSE (mm)	Mean ± SD	22 ± 4
SPAP > 30 mmHg	N (%)	8 (8)
Pericardial effusion	N (%)	11 (11)
CMR		
Active myocarditis	N (%)	77 (74)
Classic Lake Louise criteria	N (%)	49 (47)
STIR	N (%)	53 (55)
EGE	N (%)	10 (10)
LGE	N (%)	92 (88)
Abnormal T1-mapping	Fraction	35/41
Abnormal T2-mapping	Fraction	29/41
EMB		
Lymphocytic	N (%)	98 (94)
Eosinophilic	N (%)	0 (0)
Sarcoidosis	N (%)	5 (5)
Giant cell	N (%)	1 (1)
Viral genome	N (%)	18 (17)

Baseline characteristics of the population are shown. ACS = acute coronary syndrome; AF = atrial fibrillation; AVB = atrioventricular block; BA = bradyarrhythmia; CMP = cardiomyopathy; CRP = C-reactive protein; EDD = end-diastolic diameter; EDVi = end-diastolic volume (indexed); EF = ejection fraction; EGE = early gadolinium enhancement; HF = heart failure; HR = heart rate; IQR = interquartile range; LGE = late gadolinium enhancement; LV = left ventricle; n.v. = normal value; NSAT = nonsustained atrial tachyarrhythmia; NSVT = nonsustained ventricular tachycardia; PVC = premature ventricular complexes; RV = right ventricle; SCD = sudden cardiac death; SD = standard deviation; SVA = supraventricular arrhythmias; T-Tn = T troponin; TAPSE = triscupid annular plane systolic excursion; VA = ventricular arrhythmias; VF = ventricular fibrillation; VT = ventricular tachycardia; WMA = wall motion abnormality.

**Table 2:** Arrhythmia detection during follow up.

Arrhythmia Type		Total	De Novo	By Month 12	Technique		
					By Holter	By CAM	*p*
VT ^1^	Patients, N (%)	45	10 (22)	25 (56)	4 (9)	45 (100)	<0.001
	Episodes, N (%)	115	-	44 (38)	5 (4)	115 (100)	-
NSVT	Patients, N (%)	67	27 (40)	44 (66)	45 (67)	64 (95)	<0.001
	Episodes, N (%)	3224	-	1515 (47)	386 (12)	2933 (91)	-
PVC	Patients, N (%) >10^3^ daily	102 71	2 (2) 4 (6)	99 (97) 66 (93)	102 (100) 71 (70)	21 (21) -	<0.001 -
AF ^2^	Patients, N (%) >24 h	19 6	13 (68) 6 (100)	7 (37) 2 (33)	3 (16) 0 (0)	19 (100) 6 (100)	<0.001 0.002
	Episodes, N (%) > 24 h	45 12	- -	9 (20) 2 (17)	4 (9) 0 (0)	45 (100) 12 (100)	- -
Atrial flutter/AT ^2^	Patients, N (%) >24 h	11 3	10 (91) 2 (67)	4 (36) 1 (33)	5 (45) 1 (33)	11 (100) 3 (100)	0.012 0.400
	Episodes, N (%) > 24 h	58 4	- -	13 (22) 1 (25)	10 (17) 1 (25)	58 (100) 4 (100)	- -
NSAT ^3^	Patients, N (%)	43	38 (88)	20 (47)	17 (40)	43 (100)	<0.001
	Episodes, N (%)	162	-	33 (20)	38 (23)	162 (100)	-
BA ^4^	Patients, N (%)	6	4 (67)	3 (50)	1 (14)	6 (100)	0.015
	Episodes, N (%)	10	-	4 (40)	1 (10)	9 (90)	-
Pause 2–3 s	Patients, N (%)	18	14 (78)	11 (61)	18 (100)	0 (0)	<0.001
	Episodes, N (%)	24	-	12 (50)	24 (100)	0 (0)	-

Arrhythmia types documented during follow up are shown as detected by Holter ECG vs. CAM. Both the number of episodes and the number of patients are reported: ^1^ VT includes sustained VT and appropriate ICD therapy (either ATP or shock); ^2^ AF and AT only include episodes lasting > 30 s; ^3^ NSAT includes supraventricular arrhythmia episodes lasting ≤ 30 s; ^4^ BA includes 2nd type II, 2:1 or 3rd degree atrioventricular blocks and pauses > 3 s. AF = atrial fibrillation (paroxysmal); AT = atrial tachycardia; ATP = anti-tachycardia pacing; BA = bradyarrhythmia; CAM = continuous arrhythmia monitoring; ICD = implantable cardioverter defibrillator; NSAT = nonsustained atrial tachyarrhythmia; NSVT = nonsustained ventricular tachycardia; PVC = premature ventricular complex; VT = ventricular tachycardia.

**Table 3:** Characteristics of primary prevention CAM patients with follow-up VT vs. without follow-up VT.

	Units	VT+ *N* = 10	VT− *N* = 47	*p*
Putative risk factors				
LVEF < 35%	N (%)	3 (30)	15 (32)	1.000
Granulomatous	N (%)	1 (10)	1 (2)	0.323
2nd/3rd degree AVB	N (%)	1 (10)	5 (11)	1.000
Fast/recurrent NSVT	N (%)	1 (10)	4 (9)	1.000
Positive PVS	N (%)	1 (10)	0 (0)	0.174
Extensive LGE or fibrosis *	N (%)	3 (30)	18 (38)	0.730
Other baseline features				
Sex (male)	N (%)	8 (80)	32 (68)	0.706
Age > 40 year	N (%)	6 (60)	26 (55)	1.000
SVA	N (%)	2 (20)	3 (6)	0.208
LVEF < 50%	N (%)	7 (70)	19 (40)	0.160
Anteroseptal LGE	N (%)	5 (50)	6 (13)	0.016
Chronically active myocarditis	N (%)	9 (90)	23 (49)	0.031

Characteristics of the 10 patients experiencing their first VT episode (VT+) during follow up are shown. Significant differences are evidenced in bold. * The definition includes extensive areas of LGE (>1 left ventricular wall, or >5 of 17 left ventricular segments) at cardiac magnetic resonance, or replacement fibrosis in >50% of endomyocardial samples undergoing histological analysis. AVB = atrioventricular blocks; CAM = continuous arrhythmia monitoring; ILR = implantable loop recorder; LGE = late gadolinium enhancement; LVEF = left ventricular ejection fraction; NSVT = nonsustained ventricular tachycardia; PVS = programmed ventricular stimulation; VT = ventricular tachycardia.

## Data Availability

Data will be made available, upon reasonable request, by emailing the correspondent author.

## Supplementary Materials

The following are available online at https://www.mdpi.com/article/10.3390/jcm10215142/s1, Supplementary Materials. Table S1: Indications to different CAM types; Table S2: Programmed ventricular stimulation; Table S3: Treatment; Table S4: Quality of life; Table S5: Arrhythmia detection according to CAM type and indication; Table S6: Clinical impact of arrhythmia detection; Figure S1: Arrhythmic event rates; Figure S2: VA modifications during follow-up.

## Abbreviations

AAD	antiarrhythmic drug
AF	atrial fibrillation
AVB	atrioventricular block
BA	bradyarrhythmia
CAM	continuous arrhythmia monitoring
CMR	cardiac magnetic resonance
EMB	endomyocardial biopsy
FU	follow up
ICD	implantable cardioverter defibrillator
ILR	implantable loop recorder
LGE	late gadolinium enhancement
LVEF	left ventricular ejection fraction
NSAT	nonsustained atrial tachyarrhythmia
NSVT	nonsustained ventricular tachycardia
PVC	premature ventricular complexes
PVS	programmed ventricular stimulation
SVA	supraventricular arrhythmias
VA	ventricular arrhythmias
VT	ventricular tachycardia
