# Supplementary material for: Continuous Electrical Monitoring in Patients with Arrhythmic Myocarditis: Insights from a Referral Center
Source: J Clin Med. 2021 Nov 1;10(21):5142. doi: 10.3390/jcm10215142 (PMC8584651; doi:10.3390/jcm10215142)
Supplement: Supplementary file 1 [file jcm-10-05142-s001.zip › jcm-1420454-supplementary.pdf]

Continuous Electrical Monitoring in Patients with Arrhythmic Myocarditis: insights from a referral center - Supplementary Material

CONTENT

1. Full Abbreviation List
2. Supplementary Methods
3. Supplementary Tables
4. Supplementary Figures
5. Supplementary References

FULL ABBRAVIATION LIST

AF= atrial fibrillation; AVB=atrioventricular blocks; BA= bradyarrhythmias; CAM= continuous arrhythmia monitoing; CL=cycle length; CMR=cardiac magnetic resonance; EGE=early gadolinium enhancement; EMB=endomyocardial biopsy; ES=electrical storm; FU=follow-up; ICD=implantable cardioverter defibrillator; ILR=implantable loop recorder; LGE=late gadolinium enhancement; NSAT=nonsustained atrial tachyarrhythmia; NSVT=nonsustained ventricular tachycardia; PCR= polymerase chain reaction; PRF=putative risk factor; PVC=premature ventricular complexes; PVS=programmed ventricular stimulation; STIR= short-tau inversion-recovery; SVA=supraventricular arrhythmias; VA=ventricular arrhythmias; VF=ventricular fibrillation; VT=ventricular tachycardia.

SUPPLEMENTARY METHODS

Definition of arrhythmias

Arrhythmias were classified based on guideline-supported definitions (1-3). ***Ventricular fibrillation*** (VF): a chaotic rhythm defined on the surface ECG by undulations that are irregular in both timing and morphology, without discrete QRS complexes (1). ***Ventricular tachycardia*** (VT): a tachycardia (rate >100 bpm) with 3 or more consecutive beats that originates from the ventricles independent of atrial or AV nodal conduction (1). Sustained VT: continuous VT for 30 seconds, or which requires an intervention for termination (iv antiarrhythmic drugs, anti-tachycardia pacing, cardioversion/shock, ablation). Nonsustained VT (NSVT): terminates spontaneously within 30 seconds. During follow-up (FU), VT included also appropriate implantable cardioverter defibrillator (ICD) therapy, including anti-tachycardia pacing or shock. ***Electrical storm*** (ES) was defined as a state of cardiac electrical instability defined by ≥3 episodes of appropriate ICD shocks within 24 h (1). ***Premature ventricular complex*** (PVC): an early ventricular depolarization with or without mechanical contraction (1). Grade ≥ 2 PVC were defined, according to the the Lown's classification, by the documentation of > 1 PVC/min or > 30 PVC/h (4). In case of > 1 PVC/min, the temporal cutoff was 30 consecutive minutes, or 60 nonconsecutive minutes. In this study, the cutoff of 30 seconds was necessary to define episodes of supraventricular arrhythmias (SVA), including all of the following variants (2). ***Atrial fibrillation*** (AF): a supraventricular arrhythmia with uncoordinated atrial activation and, consequently, ineffective atrial contraction. ECG characteristics include irregular atrial activity, absence of distinct P waves, and irregular R-R intervals (2). ***Atrial flutter***: a macroreentrant atrial tachycardia, including cavotricuspid isthmus-dependent and atypical forms.

The atrial rate is typically 300 bpm and characteristic atrial activation pattern are recognizable (2). ***Atrial tachycardia***: a focal supraventricular arrhythmia arising from a localized atrial site, characterized by regular, organized atrial activity with discrete P waves and typically an isoelectric segment between P waves (2). Episodes of SVA lasting < 30 seconds were defined as ***nonsustained atrial tachyarrhythmias*** (NSAT).

During FU, atrial tachycardia/flutter were distinguished by AF by analyzing the regularity of atial electrograms, as recorded by cardiac devices. ***Bradyarrhythmias*** (BA) included 2nd type II, 2:1, or 3rd degree atrioventricular block (AVB), and pauses secondary to either sinus bradycardia or 2nd degree type I AVB (3). For study inclusion, pauses lasting > 3 s were considered, provided the absence of drugs with negative chronotropic effect.

Characterization of arrhythmias

Information analyzed about PVC included: 1) daily number; 2) 12-lead ECG morphology: monomorphic (single PVC morphology, or one dominant representing ≥ 75% of total morphologies), or polymorphic (all of the remaining cases); for each patient, the dominant 12-leads morphology was analyzed in this study. Information collected about NSVT and VT episodes included: 1) morphology: monomorphic (same beat-to-beat morphology within the same arrhythmic episode) or polymorphic (change in beat-to-beat morphology during the same arrhythmic episode); 2) beat-to-beat regularity: irregular (cycle length =CL varying by > 40 ms, as previously defined), or regular (in the remaining cases) (5,6); at baseline assessment, beat-to-beat CL was measured from 12-leads ECG by using manual calipers with an accuracy of 40 ms. During FU, beat-to-beat CL was recorded by cardiac device electrograms.

Device programming

Except for S-ICDs, all devices had the minimal VA detection set at 150 bpm × 5 consecutive beats. As per standard ICD programming, two therapy zones were used (> 170-180 bpm for VT; > 200-220 bpm for VF), with patient-tailored adjustment restricted to secondary prevention cases. Irrespective of the device sensitivity, SVA were reported as episodes if lasting > 30 seconds, and nonsustained tachyarrhythmias (NSAT) otherwise. A 3-second interval was the treshold for pause detection in ILR carriers. All data from arrhythmia monitoring were manually reviewed by three clinical electrophysiologists (GT for in-hospital telemonitoring, GP for Holter ECGs, and AM for CAM), blinded to the study design and results.

Device programming for continuous arrhythmia monitoring (CAM) is reported in the table below.

Arrhythmia type		Device settings	ILR	Dual chamber ICD	Single chamber ICD	S-ICD
VA	VT, NSVT detection	LDR (bpm)<br>MBN (N)	150<br>5	150<br>5	150<br>5	170<br>15
	VT treatment[1]	VT1 LDR, PP (bpm)<br>VF LDR, PP (bpm)	-<br>-	170-180<br>200-220	170-180<br>200-220	170-180<br>200-220
SVA[2]	AF	LDR (bpm)<br>MD (s)	100<br>1	100<br>1	100<br>1	100<br>360
	AT, AFlu	LDR (bpm)<br>MD (s)	100<br>1	100<br>1	100<br>1	100<br>360
BA[3]	BA	Bradycardia MD<br>Bradycardia MBN (N)<br>Pause MD (s)	2<br>4<br>3	-<br>-<br>-	-<br>-<br>-	-<br>-<br>-

[1] For secondary prevention, ICD therapy programming was patient-tailored.

[2] Except for S-ICD, all devices were capable of detecting SVA episodes lasting ≥ 1 seconds. However, SVA episodes lasting ≤ 30 s were included among NSAT, and only SVA episodes lasting > 24 hours were considered as clinically relevant. In S-ICDs, 6 minutes of arrhythmia (either consecutive or cumulative in one day) were needed to diagnose SVA. To avoid inappropriate ICD shocks, all devices had the atrial high rate episodes (AHRE) discriminators activated (allowing differential diagnosis from VA, depending on onset, stability, A:V ratio, and regularity).
[3] BA were detectable and classified by ILR only. In transvenous ICD carriers, a pacemaker-dependency status was the only way to diagnose BA. In S-ICD carriers, BA were not evaluable.

PVS

Invasive programmed ventricular stimulation (PVS) was performed for arrhythmic risk stratification in 61 patients. Indication was clinically-driven, as previously described (7). PVS protocol was standard, from right femoral vein percutaneous access. We used a single pacing site at right ventricular apex. We delivered trains of 8 ventricular stimuli (S1S1 500 and 400 ms) with one, two and three extrastimuli (S2, S3, S4). We adopted decrement intervals of 20 ms from 400 to 300 ms, and of 10 ms from 300 to 200 ms. We interrupted PVS in the presence of any of the following results: a) PVS positivity, defined as the induction of sustained VT or VF; b) refractoriness; c) conduction at 3 extrastimuli at a minimal prematurity of 200 ms. We used no pharmacological provocative protocols during the procedure. At the time of PVS, patients were in at least 48 hour wash out from betablockers or antiarrhythmic drugs.

Endomyocardial biopsy and histology

Right ventricular endomyocardial biopsy (EMB) was performed via right internal jugular vein ascess in all patients. A Cordis® bioptome, 7 Fr sheath, was used. Under fluoroscopic and echocardiographic guidance, sampling was performed at interventricular septum. Five samples of endomyocardial tissue per patient were obtained. Tissue was analysed by histology, immunohistochemistry, and molecular biology. In detail, 4 samples were immediately fixed in 10% buffered formalin at room temperature for light microscopy and immunohistochemistry, and 1 sample was stored in RNA later tubes at room temperature for viral polymerase chain reaction (PCR). In the case of viral myocarditis, PCR was performed on blood sample too, to evaluate for coexisting viremia. EMB-proven myocarditis was defined, as currently accepted (8), by histological, immunohistochemical and molecular criteria. In detail: a) histology: Dallas criteria, defined as histological evidence of inflammatory infiltrates within the myocardium associated with myocyte degeneration and necrosis of nonischemic origin; however, we also included patients with no evidence of necrosis, but significant inflammatory infiltrates ("borderline" myocarditis); b) immunohistochemistry: presence of an abnormal inflammatory infiltrate, defined as follows: ≥ 14 leucocytes/mm2 including up to 4 monocytes/mm2 with the presence of CD 3 positive T-lymphocytes ≥ 7 cells/mm2; c) molecular biology: classification into viral forms, defined as histological evidence for myocarditis associated with positive viral PCR; and autoimmune or virus-negative myocarditis, defined as histological myocarditis with negative viral PCR. Agents responsible for toxic myocarditis (8) were also excluded by focused anamnesis. Based on epidemiology, in the absence of clinical or laboratory findings suggesting other infective etiologies, a standard panel of viruses was analyzed, including: Coxsackieviruses A and B, echoviruses, rhinoviruses, influenza A and B viruses, adenoviruses, parvovirus B19, cytomegalovirus, human herpes virus-6, Epstein-Barr virus, varicella-zoster virus, herpes simplex virus-1.

Cardiac magnetic resonance

Cardiac magnetic resonance (CMR) was performed on a 1.5 T scanner (Achieva dStream; Philips Medical Systems, Eindhoven, The Netherlands) equipped with a 32-channel phased-array coil. Functional imaging consisted of ECG-gated balanced steady state free precession cine sequences acquired during breath hold. Images were acquired accoriding to the updated recommendations (9), implemented with the evaluation of standard and, whwnever applicable, updated Lake Louise criteria (10,11). In detail, myocardial inflammation and edema were evaluated using black blood T2 short-tau inversion-recovery (STIR) images, native-T1 mapping and T2 mapping. Myocardial hyperemia was evaluated by early gadolinium enhancement (EGE), recorded 2 min after peripheral bolus injection of gadobutrol (Gd-DO3A-butrol; Gadovist 1.0; Bayer HealthCare Pharmaceuticals) at the dose of 0.15 mmol/kg of body weight. Late gadolinium enhancement (LGE) images were acquired 10 min after gadolinium injection using 2D T1 weighted segmented inversion-recovery gradient-echo sequences acquired in horizontal, vertical long axis, and short axis. The correct inversion time was determined using the Look-Locker technique. In all the 17 LV segments, LGE was deemed present only if visible in 2 orthogonal views (short-axis and long-axis views). T-mapping technology (T1 and T2 mapping analysis) was available only for the most recent subset of patients. Subsequently, systematic analysis of data was not performed in the current study.

Quality of life assessment
To investigate impact of implantable devices on the quality of lifes, the following questionnaire was administered to patients during FU.

Question 1
Which kind of device are you carrying?<br>Implantable loop recorder<br>Intracardiac pacemaker or defibrillator<br>Subcutaneous defibrillator
Question 2
How do you feel after device implant?<br>Better<br>Worse<br>As before implant
Question 3A
If you feel better, which is the most positive facor influencing your life?<br>(open field)
Question 3B
If you feel worse, which is the most negative facor influencing your life?<br>(open field)

SUPPLEMENTARY TABLES

Table S1
Indications to different CAM types

	Units	Total N=104	SP ICD N=47	PP ICD N=15	ILR N=42	p
LVEF < 35%	N (%)	19 (18)	3 (6)	**10 (67)**	6 (14)	<0.001
Granulomatous	N (%)	6 (6)	4 (9)	1 (7)	1 (2)	0.459
2nd/3rd degree AVB	N (%)	10 (10)	4 (9)	**5 (33)**	1 (2)	0.002
Fast/recurrent NSVT	N (%)	31 (30)	**26 (55)**	5 (33)	**0 (0)**	<0.001
Positive PVS*	N (%)	25 (24)	**24 (51)**	1 (7)	**0 (0)**	<0.001
Extensive LGE or fibrosis**	N (%)	53 (51)	32 (68)	11 (73)	**10 (24)**	<0.001
Number or PRFs	Mean±SD	1.4±1.0	2.0±0.9	2.2±0.6	0.5±0.4	<0.001

Indications to different CAM types are shown, according to the different baseline PRFs. PRF definitions are reported in the main text. For each PRF, the groups driving the statistical significance are enhanced in bold.
*PVS was performed in 61/104 patients (59%).
** The definition includes extensive ares of LGE (> 1 left ventricular wall, or > 5 of 17 left ventricular segments) at cardiac magnetic resonance, or replacement fibrosis in > 50% of endomyocardial samples undergoing histological analysis.
AVB=atrioventricular blocks; CAM=continuous arrhythmia monitoring; ICD=implantable cardioverter defibrillator; ILR=implantable loop recorder; LGE=late gadolinium enhancement; LVEF=left ventricular ejection fraction; NSVT=nonsustained ventricular tachycardia; PP=primary prevention; PRF=putative risk factor; PVS=programmed ventricular stimulation; SD=standard deviation; SP=secondary prevention.

Table S2
Programmed ventricular stimulation

	Units	Total (N=61)
Indication		
Main symptom		
Syncope	N (%)	28 (46)
Palpitation	N (%)	31 (51)
Other symptoms	N (%)	2 (3)
Main arrhythmia		
VF	N (%)	6 (10)
VT	N (%)	22 (36)
NSVT	N (%)	30 (49)
PVC	N (%)	3 (5)
Results		
Positive PVS	N (%)	25 (41)
Inducibility - drive train		

At 500 ms<br>At 400 ms	N (%)<br>N (%)	13 (21)<br>12 (20)
Inducibility - extrastimulus<br>At S2<br>At S3<br>At S4	 N (%)<br>N (%)<br>N (%)	 0 (0)<br>16 (26)<br>9 (15)
Induced arrhythmia<br>Monomorphic VT<br>Polymorphic VT or VF*	 N (%)<br>N (%)	 19 (31)<br>6 (10)
Management<br>ICD implant	 N (%)	 25 (41)

Indications and results of baseline PVS in N=61 patients are shown.
*No patients had VF induced at S4. Clinically, no VF episodes were considered as nonspecific.
ICD=implantable cardioverter defibrillator; NSVT=nonsustained ventricular tachycardia; PVC=premature ventricular complexes; PVS=programmed ventricular stimulation; S2-S4 (first-third extrastimulus); VF=ventricular fibrillation; VT=ventricular tachycardia.

Table S3
Treatment

	Units	Total N=104
Circulatory support		
Inotropes	N (%)	8 (8)
IABP	N (%)	6 (6)
Impella	N (%)	2 (2)
VA ECMO	N (%)	1 (1)
Medical treatment		
ACE-inhibitors	N (%)	77 (74)
ARB	N (%)	10 (10)
Betablockers	N (%)	96 (92)
Calcium-channel blockers	N (%)	5 (5)
Diuretics	N (%)	42 (40)
Oral anticoagulants	N (%)	11 (11)
Antiarrhythmics		
Amiodarone	N (%)	32 (31)
Sotalol	N (%)	21 (20)
Flecainide	N (%)	5 (5)
Propaphenone	N (%)	2 (2)
Mexiletine	N (%)	6 (6)
Lidocaine	N (%)	6 (6)
Multiple drugs	N (%)	23 (22)
Etiology-driven therapy		
Antiviral agents	N (%)	2 (2)
Immunosuppressive agents*	N (%)	61 (59)

Tretament during hospitalization is shown.
*Immunosuppressive agents included combination oral drug regimen (1 mg/kg prednisone and 2 mg/kg azathioprine in 51/61 patients; other immunosuppressants in the remaining 10/61 patients) for a 12-month average treatment duration.
ACE=angiotensin converting enxyme; ARB=angiotensin receptor blockers; IAPB=intra-aortic balloon pum; VA ECMO=venoarterial extracorporeal membrane oxygenator.

Table S4
Quality of life

	Units	Total	Subgroups			
		N=104	ILR N=42	TV-ICD N=53	S-ICD N=9	p
Available answers	N	100	42	50	8	
General impact						
Feels better	N (%)	41 (41)	15 (36)	22 (44)	4 (50)	0.939
Feels worse	N (%)	9 (9)	2 (5)	6 (12)	1 (13)	0.451
Feels unchanged	N (%)	50 (50)	25 (60)	22 (44)	3 (38)	0.254
Positive features						
Feels protected/controlled	N (%)	27 (27)	6 (14)	18 (36)	3 (38)	0.511
Feels reassured about symptoms	N (%)	11 (11)	8 (19)	3 (6)	0 (0)	0.071
Other	N (%)	3 (3)	1 (2)	1 (2)	1 (13)	0.258
Negative features						
Feels restricted/controlled	N (%)	3 (3)	2 (5)	1 (2)	0 (0)	0.627
Feels impaired in daily life	N (%)	3 (3)	0 (0)	3 (6)	0 (0)	0.509
Feels pain/discomfort	N (%)	2 (2)	0 (0)	1 (2)	1 (13)	0.417
Other	N (%)	1 (1)	0 (0)	1 (2)	0 (0)	1.000

Results of the quality of life questionnaire are shown for all device carriers and subgroups.
ICD=implantable cardioverter defibrillator; ILR=implantable loop recorder; S=subcutaneous; TV=transvenous.

Table S5
Arrhythmia detection according to CAM type and indication

Arrhythmia type		Total	Total By CAM	By ICD N=62	By ILR N=42	p	By SP ICD N=47	By PP CAM N=57	p
VT[1]	Patients, N (%)	45	45 (100)	43 (69)	2 (5)	<0.001	35 (74)	10 (18)	<0.001
	Episodes, N (%)	115	115 (100)	113 (98)	2 (2)	-	103 (90)	12 (10)	-
NSVT	Patients, N (%)	67	64 (95)	44 (71)	23 (55)	0.033	36 (77)	31 (54)	0.024
	Episodes, N (%)	3224	2933 (91)	1817 (56)	1116 (35)	-	1730 (54)	1203 (37)	-
PVC	Patients, N (%)	102	21 (21)	13 (21)	8 (19)	1.000	11 (23)	10 (18)	0.474
	$>10^3$ daily	71	-	-	-	-	-	-	-
AF[2]	Patients, N (%)	19	19 (100)	11 (18)	8 (19)	1.000	10 (21)	9 (16)	0.611
	>24 hours	6	6 (100)	2 (3)	4 (10)	0.218	1 (2)	5 (9)	0.218

	Episodes, N (%)	45	45 (100)	29 (64)	16 (36)	-	25 (55)	20 (45)	-
	> 24 hours	12	12 (100)	7 (58)	5 (42)	-	6 (50)	6 (50)	-
Atrial flutter /AT[2]	Patients, N (%)	11	11 (100)	6 (10)	5 (12)	0.753	4 (9)	7 (12)	0.750
	>24 hours	3	3 (100)	2 (3)	1 (2)	1.000	1 (2)	2 (4)	1.000
	Episodes, N (%)	58	58 (100)	35 (60)	23 (40)	-	29 (50)	29 (50)	-
	> 24 hours	4	4 (100)	2 (50)	2 (50)	-	1 (25)	3 (75)	-
NSAT[3]	Patients, N (%)	43	43 (100)	26 (42)	17 (40)	1.000	22 (47)	21 (37)	0.324
	Episodes, N (%)	162	162 (100)	84 (52)	78 (48)	-	73 (45)	89 (55)	-
BA[4]	Patients, N (%)	6	6 (100)	1 (2)	5 (12)	0.038	1 (2)	5 (9)	0.218
	Episodes, N (%)	10	9 (90)	1 (10)	9 (90)	-	1 (10)	9 (90)	-

Arrhythmia types documented during follow-up are shown, as detected by different CAM types and indications. Both number of episodes and number of patients are reported.

[1] VT include sustained VT and appropriate ICD therapy (either ATP or shock).

[2] AF, atrial flutter and AT include only episodes lasting > 30 seconds.

[3] NSAT include supraventricular arrhythmia episodes lasting ≤ 30 seconds.

[4] BA include 2nd type II, 2:1 or 3rd degree atrioventricular blocks, and pauses > 3 s.

AF= atrial fibrillation (paroxysmal); AT=atrial tachycardia; ATP=anti-tachycardia pacing; BA=bradyarrhythmias; CAM=continuous arrhythmia monitoring; ICD=implantable cardioverter defibrillator; ILR=implantable loop recorder; NSAT= nonsustained atrial tachyarrhythmia; NSVT=nonsustained ventricular tachycardia; PP=primary prevention; PVC=premature ventricular complex; SP=secondary prevention; VT=ventricular tachycardia.

Table S6
Clinical impact of arrhythmia detection

Arrhythmia type		Tot	Strategy modification			AAD/BB DA	AAD change	dual-ch. ICD upgrade	TCA	OAC
			By Holter	By CAM	p					
VT[1]	All, N (%)	45	4 (9)	41 (91)	<0.001	8 (18)	37 (82)	2 (4)	30 (65)	0 (0)
	AS, N (%)	3	3 (100)	3 (100)	1.000	0 (0)	0 (0)	0 (0)	3 (100)	0 (0)
NSVT	All, N (%)	67	10 (15)	48 (72)	<0.001	29 (43)	26 (39)	3 (4)	4 (6)	0 (0)
	AS, N (%)	43	3 (7)	30 (70)	<0.001	17 (40)	15 (35)	1 (2)	0 (0)	0 (0)
PVC	All, N (%)	102	56 (55)	8 (8)	<0.001	36 (35)	28 (27)	0 (0)	7 (7)	0 (0)
	AS, N (%)	67	29 (43)	3 (4)	<0.001	19 (28)	10 (15)	0 (0)	1 (1)	0 (0)
AF[2]	All, N (%)	19	0 (0)	9 (47)	0.001	0 (0)	1 (5)	3 (16)	1 (5)	6 (32)
	AS, N (%)	13	0 (0)	4 (31)	0.096	0 (0)	0 (0)	0 (0)	0 (0)	4 (31)
Atrial flutter/AT[2]	All, N (%)	11	1 (9)	2 (18)	1.000	0 (0)	0 (0)	0 (0)	2 (18)	2 (18)
	AS, N (%)	8	1 (9)	2 (18)	1.000	0 (0)	0 (0)	0 (0)	2 (18)	2 (18)
NSAT[3]	All, N (%)	43	0 (0)	0 (0)	1.000	0 (0)	0 (0)	0 (0)	0 (0)	0 (0)
	AS, N (%)	40	0 (0)	0 (0)	1.000	0 (0)	0 (0)	0 (0)	0 (0)	0 (0)
BA[4]	All, N (%)	6	1 (17)	5 (83)	0.080	5 (83)	2 (33)	3 (4)	0 (0)	0 (0)
	AS, N (%)	4	1 (25)	4 (100)	0.143	4 (100)	1 (25)	1 (2)	0 (0)	0 (0)
Pause 2-3 s	All, N (%)	18	1 (6)	0 (0)	1.000	1 (6)	0 (0)	0 (0)	0 (0)	0 (0)
	AS, N (%)	18	1 (6)	0 (0)	1.000	1 (6)	0 (0)	0 (0)	0 (0)	0 (0)

Modifications in arrhythmia management strategies are shown, as guided by Holter ECG and CAM findings during follow-up. Data are shown both for the whole cohort, and for asymptomatic patients.

[1] VT include sustained VT and appropriate ICD therapy (either ATP or shock).

[2] AF, atrial flutter and AT include only episodes lasting > 30 seconds.

[3] NSAT include supraventricular arrhythmia episodes lasting ≤ 30 seconds.

[4] BA include 2nd type II, 2:1 or 3rd degree atrioventricular blocks, and pauses > 3 s.

AAD=antiarrhythmic drugs; AF= atrial fibrillation (paroxysmal); AS=asymptomatics; AT=atrial tachycardia; ATP=anti-tachycardia pacing; BA=bradyarrhythmias; BB=betablockers; CAM=continuous arrhythmia monitoring; DA=dose-adjustment; ICD=implantable cardioverter defibrillator; ILR=implantable loop recorder; NSAT= nonsustained atrial tachyarrhythmia; NSVT=nonsustained ventricular tachycardia; OAC=oral anticoagulants; PP=primary prevention; PVC=premature ventricular complex; SP=secondary prevention; TCA=transcatheter ablation; VT=ventricular tachycardia.

SUPPLEMENTARY FIGURES

Figure S1
Arrhythmic event rates

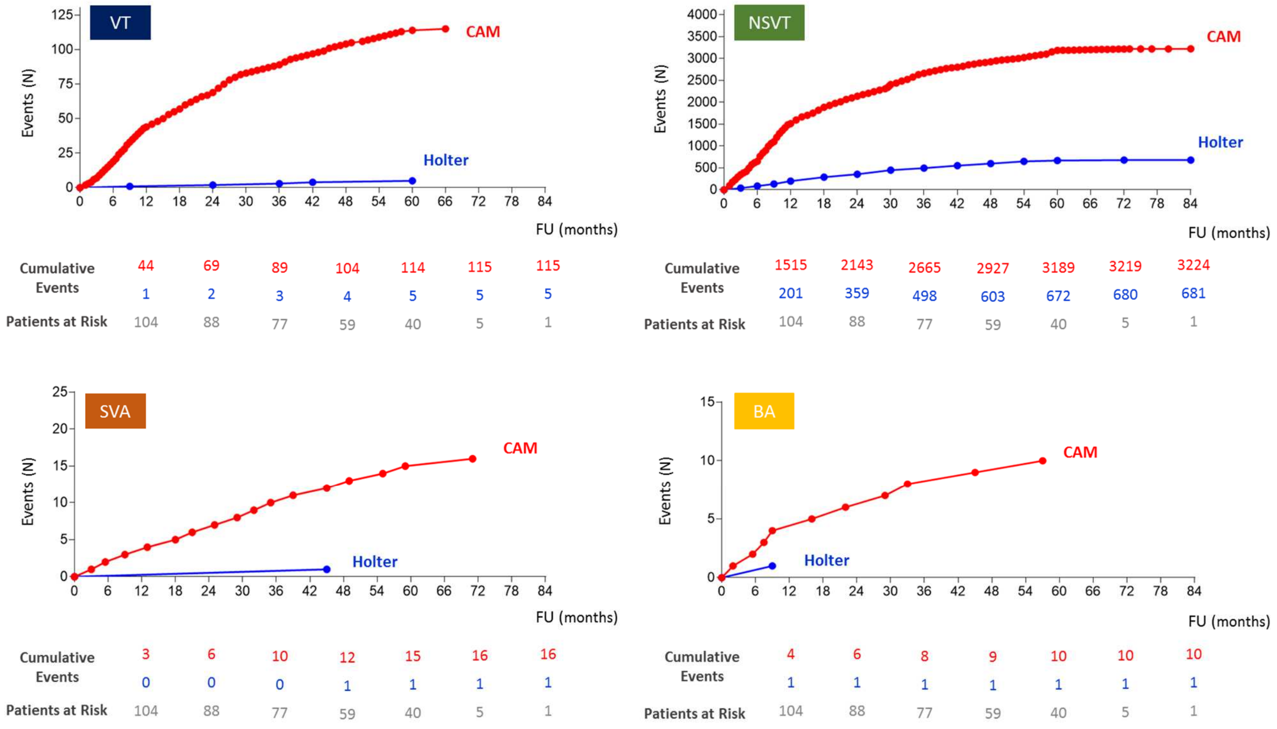

Cumulative event rates are shown for the endpoints of VT, NSVT, SVA and BA, as documented by CAM (red) vs. sequential 24h Holter ECGs (blue).

BA=bradyarrhythmias; CAM= continuous arrhythmia monitoing; FU=follow-up; Holter=24h Holter ECG; NSVT=nonsustained ventricular tachycardia SVA=supraventricular arrhythmias; VT=ventricular tachycardia.

Figure S2
VA modifications during follow-up

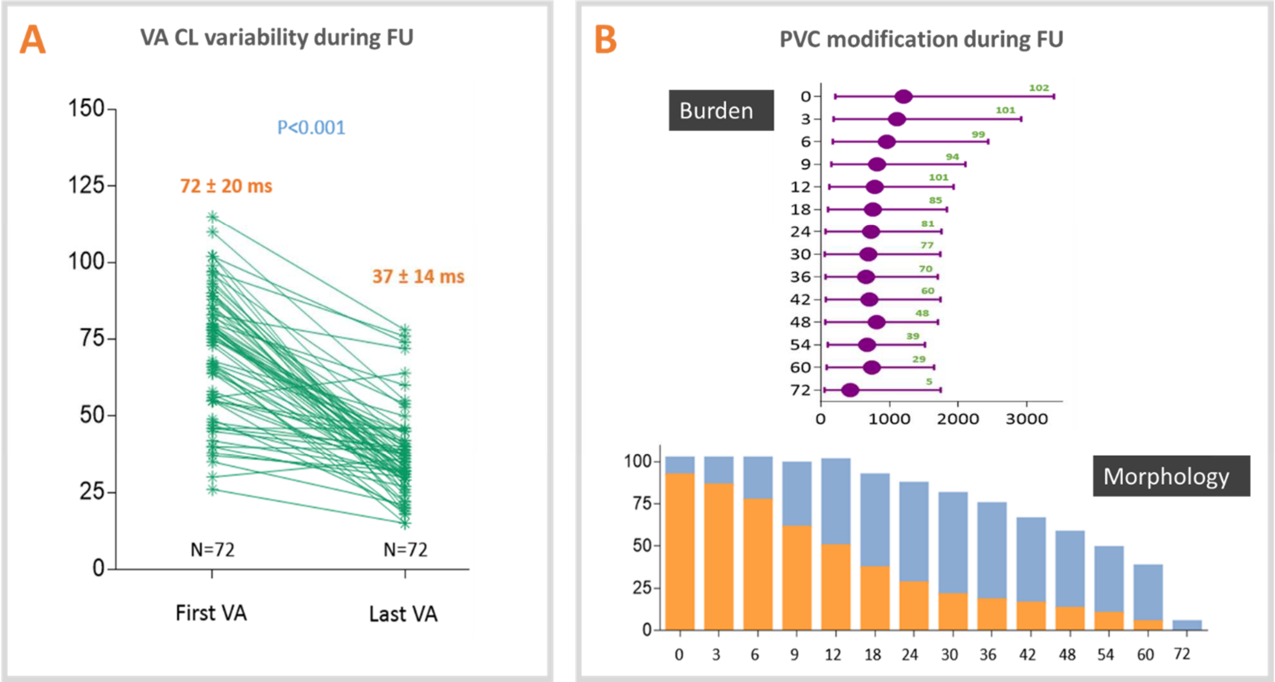

Panel A. Modification of CL variability during FU, in n=72 ptients with recurrent VA (including VT and NSVT). Maximal CL variability (in ms), measured at the difference between the maximal and the minimal CL intervals detected by CAM within the same episode, is plotted on the y-axis. Panel B. Upper panel: variation in daily PVC burden as documented by sequential 24h Holter ECGs FU (in months, on the y-axis). At each timepoint, median and interquartile range are shown for the daily PVC distribution, with the total number of 24h Holter ECGs analyzed reported in green. Lower panel: modification of PVC morphology, as recorderd by baseline telemonitoring and sequential Holter ECGs during FU. FU timeline (in months) is plotted on the x-axis. The number of available Holter ECGs is plotted on the y-axis. Orange bars represent the proportion of polymorphic PVCs, whereas blue bars indicate monomorphic PVCs.
CAM= continuous arrhythmia monitoing; CL=cycle length; FU=follow-up; PVC=premature ventricular complexes; VA=ventricular arrhythmias.

SUPPLEMENTARY REFERENCES

1. Cronin EM, Bogun FM, Maury P, et al. 2019 HRS/EHRA/APHRS/LAHRS expert consensus statement on catheter ablation of ventricular arrhythmias: executive summary. Europace. 2020 Mar 1;22(3):450-495. doi: 10.1093/europace/euz332.
2. Page RL, Joglar JA, Caldwell MA, et al. 2015 ACC/AHA/HRS Guideline for the Management of Adult Patients With Supraventricular Tachycardia: A Report of the American College of Cardiology/American Heart Association Task Force on Clinical Practice Guidelines and the Heart Rhythm Society. J Am Coll Cardiol. 2016; 67:e27-e115.
3. Brignole M, Auricchio A, Baron-Esquivias G, et al. 2013 ESC guidelines on cardiac pacing and cardiac resynchronization therapy: the task force on cardiac pacing and resynchronization

therapy of the European Society of Cardiology (ESC). Developed in collaboration with the European Heart Rhythm Association (EHRA). Europace. 2013;15:1070-118.

4. Lown B, Wolf M. Approaches to sudden death from coronary heart disease. Circulation 1971;44:130-142.
5. Oreto G, Satullo G, Luzza F, Donato A, Scimone IM, Cavalli A. Irregular ventricular tachycardia: a possible manifestation of longitudinal dissociation within the reentry pathway. Am Heart J 1992;124:1506-1511.
6. Peretto G, Sala S, Rizzo S, et al. Ventricular Arrhythmias in Myocarditis: Characterization and Relationships With Myocardial Inflammation. J Am Coll Cardiol 2020;75:1046-1057.
7. Peretto G, Sala S, Basso C, Della Bella P. Programmed ventricular stimulation in patients with active vs previous arrhythmic myocarditis. J Cardiovasc Electrophysiol 2020;31:692-701.
8. Caforio AL, Pankuweit S, Arbustini E, et al. Current state of knowledge on aetiology, diagnosis, management, and therapy of myocarditis: a position statement of the European Society of Cardiology Working Group on Myocardial and Pericardial Diseases. Eur Heart J 2013;34:2636-2648.
9. Kramer CM, Barkhausen J, Flamm SD, et al. Standardized cardiovascular magnetic resonance imaging protocols, society for cardiovascular magnetic resonance: board of trustees task force on standardized protocols. Journal of Cardiovascular Magnetic Resonance 2008, 10:35.
10. Friedrich MG, Sechtem U, Schulz-Menger J, et al.; International Consensus Group on Cardiovascular Magnetic Resonance in Myocarditis. Cardiovascular magnetic resonance in myocarditis: A JACC White Paper. J Am Coll Cardiol 2009;53:1475-87.
11. Ferreira VM, Schulz-Menger J, Holmvang G, at al. Cardiovascular Magnetic Resonance in Nonischemic Myocardial Inflammation: Expert Recommendations. J Am Coll Cardiol. 2018;72:3158-3176.
